# Production of a Lipopeptide Biosurfactant by a Novel *Bacillus* sp. and Its Applicability to Enhanced Oil Recovery

**DOI:** 10.1155/2013/621519

**Published:** 2013-09-24

**Authors:** Thivaharan Varadavenkatesan, Vytla Ramachandra Murty

**Affiliations:** Department of Biotechnology, Manipal Institute of Technology, Manipal University, Manipal, Karnataka 576104, India

## Abstract

Biosurfactants are surface-active compounds derived from varied microbial sources including bacteria and fungi. They are secreted extracellularly and have a wide range of exciting properties for bioremediation purposes. They also have vast applications in the food and medicine industry. With an objective of isolating microorganisms for enhanced oil recovery (EOR) operations, the study involved screening of organisms from an oil-contaminated site. Morphological, biochemical, and 16S rRNA analysis of the most promising candidate revealed it to be *Bacillus siamensis*, which has been associated with biosurfactant production, for the first time. Initial fermentation studies using mineral salt medium supplemented with crude oil resulted in a maximum biosurfactant yield of 0.64 g/L and reduction of surface tension to 36.1 mN/m at 96 h. Characterization studies were done using thin layer chromatography and Fourier transform infrared spectroscopy. FTIR spectra indicated the presence of carbonyl groups, alkyl bonds, and C–H and N–H stretching vibrations, typical of peptides. The extracted biosurfactant was stable at extreme temperatures, pH, and salinity. Its applicability to EOR was further verified by conducting sand pack column studies that yielded up to 60% oil recovery.

## 1. Introduction 

Biosurfactants are amphiphilic molecules which have the ability to depict a wide variety of surface activity. They comprise both a hydrophobic and a hydrophilic group that aid in its accumulation between fluid phases. Biosurfactants have the natural tendency to decrement surface and interfacial tension. Because of this property, they allow easy accessibility to nonpolar hydrocarbons so that microorganisms in oil-rich ecological niches can easily degrade them.

A number of high molecular weight biosurfactants and bioemulsifiers are produced by both bacteria and fungi. Biosurfactants of bacterial origin belong to most classes of compounds including polysaccharides, proteins, lipopolysaccharides, lipoproteins and combinations of many of these structural types. Bacterial strains belonging to the genus *Bacillus* and *Pseudomonas* usually produce lipopeptide biosurfactants. Almost all classes of microorganisms (Table 1) produce biosurfactants (Finnerty [1] and Healy et al. [2]). 

Owing to their xenobiotic nature, synthetic surfactants have the potential disadvantage of persisting in the environment, long after they are applied for a remedial measure. Also, some of the synthetic surfactants are comparatively more toxic to human health (Dehghan-Noudeh et al. [3]). As biosurfactants are of microbial origin, they have been under the active scrutiny of researchers for more than a decade. Biosurfactants have the potential to be considered as a viable alternative to the chemically synthesized surfactants for environmental cleanup.

According to Habe and Omori [4], the biological treatment of PAH-contaminated soil should be an economically viable and efficient process. The biological approach has a lot of advantages including complete degradation of the pollutants, lower treatment cost, greater safety, and lesser soil disturbance. Kosaric [5] enlisted an exhaustive list of advantages in favour of biosurfactants: biodegradability, low toxicity, biocompatibility and digestibility, availability of raw materials for production, acceptable production economics, environmental control, specificity, and effectiveness. Biodegradation of the hydrocarbons at a specific contaminated site depends on the indigenous soil microbial population, type and concentration of hydrocarbons present, soil characteristics, and availability of nutrient and oxygen. The genera of soil microorganisms that are known to degrade hydrocarbons include *Pseudomonas, Flavobacterium, Achromobacter, Arthrobacter, Micrococcus, and Acinetobacter* (Kosaric [5]).

The present work attempts to investigate the use of a novel biosurfactant-producing bacterial strain, isolated from oil-contaminated sites in Manipal (Karnataka, India) for enhanced oil recovery operations.

## 2. Materials and Methods

### 2.1. Isolation and Enrichment of Microorganisms

Oil-soaked soil samples were collected from a local automobile workshop in Manipal (Karnataka, India). The samples were enriched by inoculating 1 g of the soil sample into 50 mL of sterile Bushnell Haas broth (HiMedia, Mumbai) [6], taken in a 250 mL conical flask at 30°C in a shaker incubator (Rotek, India), & set at 150 rpm. The medium was also constituted with filter-sterilized 1% (v/v) n-hexadecane as the sole carbon source. Serial dilution of the sample was performed after 48 h of incubation and plated onto sterile Bushnell Haas agar plates. After incubation for 48 h at 30°C, morphologically distinct colonies were reisolated by transfer to fresh agar plates thrice to obtain pure cultures. The chosen isolates were further screened for the production of biosurfactants using multiple screening methods.

### 2.2. Screening for Biosurfactant Producers

The preliminary screening assays for biosurfactant production were performed using a variety of methods. 48-hour-old cultures of the isolates grown in Bushnell Haas broth were taken to perform the screening tests. All the screening tests were performed in triplicate.

In the oil spreading technique developed by Morikawa et al. [7], 30 mL of distilled water was taken in a Petri dish to which 1 mL of coconut/sesame oil was added to the centre. 20 *μ*L of the culture supernatant from the broth was added on top of the oil layer. The Petri dishes were closely observed for a zone of displacement in the oil, and the diameter of displacement was measured.

Blood agar Hemolysis test was performed to check the hemolytic activity of the microbial isolates, as described by Mulligan et al. [8]. The isolates were streaked onto sheep blood agar plates (Himedia, Mumbai) in a sterile environment. The plates were incubated for 48–72 hours at 30°C. The bacterial colonies were visually examined for the presence of clear zones around the streaks. The extent of clearing was classified into 4 categories, as described by Rodrigues et al. [9]: no hemolysis; incomplete to partial hemolysis with a clearing <1 cm; complete hemolysis with a clearing >1 cm but <3 cm; complete hemolysis with a clearing >3 cm. 

The drop collapse test developed by Jain et al. [10] depends on the breakdown of liquid droplets due to surfactant action. Sterile glass slides were coated with commercially available engine oil (SG SAE 20W-40 grade) and fully covered to allow equilibration for 24 hours at room temperature. 0.01 mL of the culture supernatant was dropped on the surface of the equilibrated glass slides. The shape of the drops was observed for activity of the culture supernatant on the oil after an hour. Depending on the concentration of the crude biosurfactant, the drop collapses to varying degrees. The extent of drop collapse was assessed as follows: no collapse; partial collapse if diameter after collapse <1 cm; near complete collapse if diameter after collapse >1 cm and <1.5 cm; complete collapse if diameter after collapse >1.5 cm.

CTAB agar Plate test, developed by Siegmund and Wagner [11], was performed for detection of anionic surfactants. Mineral salts agar (HiMedia, Mumbai) was supplemented with 2% (w/v) glucose as carbon source, 0.5 mg/mL cetyltrimethylammonium bromide (HiMedia, Mumbai), and 0.2 mg/mL Methylene blue (HiMedia, Mumbai) as performed by Satpute et al. [12]. A well was punctured into the plate using a sterile cork borer and filled with 50 *μ*L of the culture supernatant. The plates were incubated for 48–72 hours at 30°C and observed for the appearance of bluish/greenish halos around the wells to imply biosurfactant production.

In the tilting glass slide test, developed by Persson and Molin [13], a single colony is picked up from the Bushnell Haas agar plate and transferred on the surface of a sterile glass slide near one of the edges. It is then mixed with a droplet of 1% saline. The slide is gradually tilted to the other side and was examined for flow of a water droplet over its surface. Biosurfactant production is implied if water flows over the surface.

The surface tension of the 48-hour-old culture broth was measured using a digital surface tensiometer (described in Section 2.7.2). This presents a proportional estimate of the biosurfactant produced by the isolates.

### 2.3. Bacterial Identification by Biochemical and 16S rRNA Sequencing Technique

The microbial isolate RT10 was identified based on its morphological and biochemical characteristics as per Bergey's Manual of Determinative Bacteriology [14]. Gene sequencing (16S rRNA method) was performed at Agharkar Research Institute (Pune, India) to identify the bacterial strain. Genomic DNA was isolated from the culture by using a commercial kit (GenElute Bacterial Genomic DNA Kit, Sigma, USA). A polymerase chain reaction was carried out using the universal primers for 1.5 kb fragment amplification for eubacteria. The 20 *μ*L master mix for the PCR was composed of 3 *μ*L of template DNA (10 ng), 2 *μ*L each of 200 *μ*M dNTP mix and 10X PCR buffer, 0.4 *μ*L each of forward and reverse primers, and 0.2 *μ*L of *Taq* DNA polymerase (Bangalore Genei, Bangalore) in 12 *μ*L of double distilled water. The PCR was performed using gradient Mastercycler system (Eppendorf, Germany) with the following cycle program: 94°C for 5 min; 30 cycles of 94°C, 60°C, and 72°C for 1 min each, and final extension at 72°C for 10 min followed by a final sample hold at 4°C. The PCR product was precipitated using 8.5% Polyethylene glycol—6000, washed thrice using 70% ethanol, and dissolved in 10 mM Tris-HCl (pH 8). The PCR product was then processed for cycle sequencing reaction. The samples were cleaned up and loaded on the sequencer. The ABI Prism BigDye Terminator Cycle Sequencing Ready Reaction Kit (Applied Biosystems, USA) was used for the sequencing reaction. Samples were run on Avant 3100 Gene Analyzer (Applied Biosystems, USA). The sequencing output was analyzed using the DNA sequence analyzer software (Applied Biosystems). The sequence was compared with National Center for Biotechnology Information (NCBI) GenBank entries by using the BLAST algorithm.

### 2.4. Screening for Plasmid DNA

Samples of the identified microbial isolate were obtained both from Bushnell Haas Broth and Nutrient Broth (HiMedia, Mumbai). Plasmid extraction was performed using the alkaline lysis method of Birnboim and Doly [15]. *Escherichia coli* DH5*α* cells harbouring the plasmid pUC18 were used as a positive control. The extracted plasmid DNA samples and the control were subjected to agarose gel electrophoresis (0.7% agarose) as described by Sambrouk and Russell [16]. The gel was observed for presence of bands corresponding to plasmid DNA.

### 2.5. Fermentation Using Crude Oil as Carbon Source

Nutrient broth was used for the preparation of the seed inoculum. The bacterial isolate RT10 was inoculated in 25 mL sterile nutrient broth in a shaking incubator set at 30°C and 150 rpm until OD_600 nm_ reaches 0.8-0.9. This seed culture was used in the production medium at 2% (v/v). As formulated by Makkar and Cameotra [17], biosurfactant production was carried out in 250 mL conical flasks containing 50 mL of a mineral salt medium with the following composition: KNO_3_ (0.3%), Na_2_HPO_4_ (0.22%), KH_2_PO_4_ (0.014%), NaCl (0.001%), MgSO_4_ (0.06%), CaCl_2_ (0.004%), FeSO_4_ (0.002%), and 0.1 mL of trace element solution containing (g/L): 2.32 g ZnSO_4_·7H_2_O, 1.78 g MnSO_4_·4H_2_O, 0.56 g H_3_BO_3_, 1.0 g CuSO_4_·5H_2_O, 0.39 g Na_2_MoO_4_·2H_2_O, 0.42 g CoCl_2_·6H_2_O, 1.0 g EDTA, 0.004 g NiCl_2_·6H_2_O, and 0.66 g KI (all chemicals were of analytical grade from Merck, USA). Crude oil (from a local refinery) was used as the sole source of carbon at 2% (v/v) concentration. The temperature of the medium was maintained at 30°C with shaking at 150 rpm. Culture medium samples were drawn for estimation of biomass, biosurfactant production, and surface tension, once every 24 hours for five days. Bacterial cell growth was monitored by measuring the dry cell weight, as described by Cooper and Goldenberg [18]. Biosurfactant concentration in the culture broth was estimated after its crude extraction and concentration. Surface tension was measured with a digital tensiometer. A conical flask without the crude oil was maintained as control. All the experiments were performed in triplicate.

### 2.6. Extraction of Biosurfactant

The culture broth was centrifuged in a refrigerated centrifuge (Plastocraft Model Superspin R-V/Fm, Mumbai) at 10000 rpm for 20 min at 4°C to obtain a cell-free supernatant. The pH of the supernatant was adjusted to 2 using 6N HCl and was subjected to acid precipitation by placing it at 4°C overnight. The off-white precipitate was separated by centrifugation at 10000 rpm for 30 min at 4°C. The precipitate was extracted thrice with a 2 : 1 chloroform-ethanol mixture. The organic phase was removed, and the biosurfactant was concentrated using a rotary evaporator (Superfit Model SuperVac, Mumbai) at 40°C. The solvents were evaporated leaving behind relatively pure biosurfactant as a viscous light brown matter.

### 2.7. Analytical Techniques

#### 2.7.1. Estimation of Biomass

At periodic time intervals, 1 mL samples of culture broth were collected in a sterile manner and centrifuged at 10000 rpm for 20 min. The biomass paste was washed thrice with 0.9% w/v saline solution. The paste was dried by heating in a hot air oven set at 50°C–70°C until constant weight was attained, without allowing the cells to be charred. 

#### 2.7.2. Surface Tension Measurements

The surface tension property was studied by taking a sample of the culture broth and centrifuging at 10000 rpm for 20 min. The cell pellet was discarded, and the surface tension of the supernatant was measured by the Wilhelmy plate method [19] using a Sigma Model 702 digital surface tensiometer (KSV Instruments Ltd., Helsinki, Finland). Initially, the plate and glassware were cleaned with chromic acid, Milli-Q water, and acetone. They were then flamed with a Bunsen burner. The instrument was calibrated beforehand using Milli-Q water. All the measurements were taken in triplicate. 

#### 2.7.3. EMI Measurements

The ability of the biosurfactant to emulsify hydrocarbons was determined by the addition of 2 mL sample of the culture supernatant and 2 mL of a hydrocarbon (hexadecane), taken in a glass test tube. The tube was vortexed at high speed for 5 min. The emulsification activity was checked after being allowed to settle for 24 h, and the emulsification index (E_24_) was calculated by measuring the emulsion layer, expressed as a percentage of the total height of the mixture in the tube, as described by Cooper and Goldenberg [18]. The emulsification power of a mixture of equal volumes of 1 mg/mL SDS and the hydrocarbon was used as the control.

#### 2.7.4. Thin Layer Chromatography

The biosurfactant sample was spotted on precoated silica gel 60 F_254_ plate (Merck, USA) and subjected to thin layer chromatography (TLC), as described by Das et al. [20]. The plate was developed with a solvent system consisting of chloroform, methanol, and water (65 : 25 : 4). The plate was visualized using a short-wave UV lamp. Further, the plate was sprayed with 0.2% ninhydrin (in absolute alcohol) and heated to 110°C.

#### 2.7.5. Fourier Transform Infrared Spectroscopy

To understand the overall chemical nature of the extracted biosurfactant, Fourier transform infrared spectroscopy (FTIR) was employed. The technique helps to explore the functional groups and the chemical bonds present in the crude extract. The analysis was done using Shimadzu FTIR Spectrophotometer (Model 8400S). Samples were prepared by homogeneous dispersal of 1 mg of the biosurfactant sample in pellets of potassium bromide (Merck, USA). IR absorption spectra were obtained using a built-in plotter. IR spectra were collected over the range of 450–4500 cm^−1^ with a resolution of 4 cm^−1^. The spectral data were the average of 50 scans over the entire range covered by the instrument. The spectrum was studied to interpret the chemical nature of the biosurfactant fraction.

### 2.8. Stability Analysis of the Biosurfactant

The stability studies were carried out with respect to the effect of temperature, pH, and salinity on surface tension and emulsification capacity of the biosurfactant. The analysis was done using the 24-hour cell-free culture broth obtained by centrifuging the culture sample at 10000 rpm for 15 minutes. All the experiments were carried out in triplicate. To study the effect of temperature, 10 mL of the cell-free broth was incubated at temperatures ranging from 4°C to 121°C for 30 minutes. The effect of pH was determined by estimating the variation of surface activity by adjusting the pH of 10 mL of cell-free broth from 2 to 12 with 6N HCl or 6N NaOH solutions. The effect of salinity was checked by varying the concentration of sodium chloride (0% to 20% w/v), added to 10 mL samples of cell-free broth. The contents were homogeneously mixed. In all the three studies, the samples were allowed to stand at room temperature for 6 hours after the respective treatments, before making the measurements of surface tension and emulsification index.

### 2.9. Suitability for Microbial Enhanced Oil Recovery

Microbial enhanced oil recovery (MEOR) is a unique residual oil extraction technology making use of microorganisms. The technique finds great application in enhancing the oil recovery from oil reservoirs. The suitability of the biosurfactant for MEOR was investigated by employing a jacketed glass column as described by Abu-Ruwaida et al. [21]. The performance of the biosurfactant in terms of oil recovery was also compared against sodium dodecyl sulphate (SDS) (Merck, USA), an amphiphilic surfactant and a common ingredient of many commercial detergents. 75 mg of sand was pretreated by washing with 1N HCl in a conical flask, rotated at 150 rpm for 1 hour. It was dried completely in a hot air oven (Rotek, India) set at 100°C for 12 hours. A glass column (45 cm × 2 cm i.d.) provided with an external jacket was packed with the sand. The column was saturated with 50 mL of commercially available engine oil (SL 20W-40; JASO M 345 grade). An aqueous solution of 25 mg biosurfactant in 50 mL distilled water was applied to the column. The temperature of the column was maintained at 30°C by passing water through the jacket using a peristaltic pump coupled to a water bath maintained at 30°C. The leachate from the column was collected over a 36-hour period, and the volume of engine oil released from the column was measured. The impact of temperature on biosurfactant-mediated oil recovery was observed by conducting similar runs at 50°C and 70°C. In a separate set of studies, an aqueous solution of 25 mg SDS in 50 mL sterile water was poured onto the column and the oil recovery was determined at 30°C, 50°C, and 70°C.

## 3. Results and Discussion

### 3.1. Isolation and Enrichment of Microorganisms

A total of 11 different bacterial specimens were isolated from the oil contaminated soil samples. The isolates were chosen based on their distinct colony morphology, obtained by serial dilution and streak plating techniques.

### 3.2. Screening for Biosurfactant Producers

The eleven isolates were then subjected to screening for biosurfactant production by multiple methods like Oil spreading technique, blood agar haemolysis, drop collapse test, CTAB agar plate test, and tilting glass slide test (Table 2).

In the oil spreading technique, Morikawa et al. [7] showed that the extent of oil displacement is directly proportional to the concentration of the biosurfactant produced. Of the eleven isolates, four samples significantly displaced the oil layer and started to spread in the water, showing a zone of displacement. In the blood agar technique, Mulligan et al. [8] relied on the hemolytic activity of the biosurfactant producers. However, it is not necessary that all biosurfactants have a hemolytic activity (Carrillo et al. [22]). Rodrigues et al. [9] scored the hemolytic activity. Accordingly, in the present study, three of the isolates displayed excellent hemolytic activity. Jain et al. [10] described the drop collapse test according to which the degree of collapse of the culture supernatant describes the surfactant concentration. Of the eleven isolates, three strains showed near-complete collapse, while for two other samples the drops turned absolutely flat. In the CTAB test designed by Siegmund and Wagner [11], two isolates showed greenish halos around the colonies on CTAB methylene blue agar medium. The tilted glass slide test, developed by Persson and Molin [13], was positive for four isolates. Water flowed over the slides on which these cultures were tested.

By experiment, none of the isolates showed positive test results for all the six screening procedures. But in the case of RT10, the rate of drop collapse was very rapid and exhibited the lowest surface tension of 29.8 mN/m. It satisfied four of the six tests, and hence, this isolate was picked up as a potential candidate for further studies. The current report, henceforth, discusses the potential use of the strain RT10 for enhanced oil recovery. 

### 3.3. Morphological, Biochemical, and 16S rRNA Analysis

The results of screening procedures consistently showed the biosurfactant-producing property of the isolate. The results of almost all the tests led to the selection of the bacterial isolate, RT10. The reduction of surface tension was the greatest in case of RT10. Based on morphological and biochemical analysis, in accordance with Bergey's Manual of Determinative Bacteriology [14], the best isolate belonged to the genus *Bacillus *(Tables 3 and 4).

**Figure 1:**
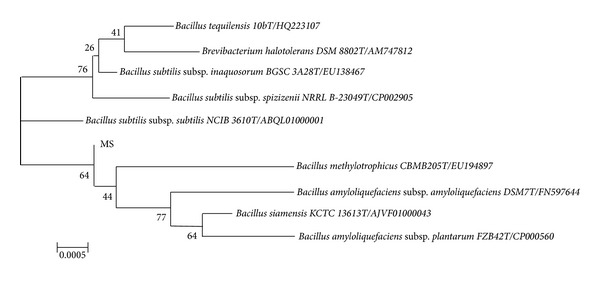
The neighbor-joining tree based on the 16S rRNA sequence, demonstrating the phylogenetic position of strain RT10.

The 16S rRNA analysis revealed that the isolate RT10 showed 99.8% similarity to *Bacillus siamensis*. The neighbor-joining tree based on the 16S rRNA sequence for the strain has been shown in Figure 1. The 16S rRNA sequence alignment shows that the strain RT10 was closely related to the species in genus *Bacillus*.

### 3.4. Screening for Plasmid DNA

The results of the plasmid extraction process did not reveal the presence of any plasmid in the isolate, irrespective of the media in which the cells were cultured. The study infers that the ability of the isolate to produce biosurfactant was not conferred upon due to the presence of any plasmid. The property should be the result of a chromosomally-mediated mechanism of the bacterium.

### 3.5. Fermentation Using Crude Oil as Carbon Source

**Figure 2:**
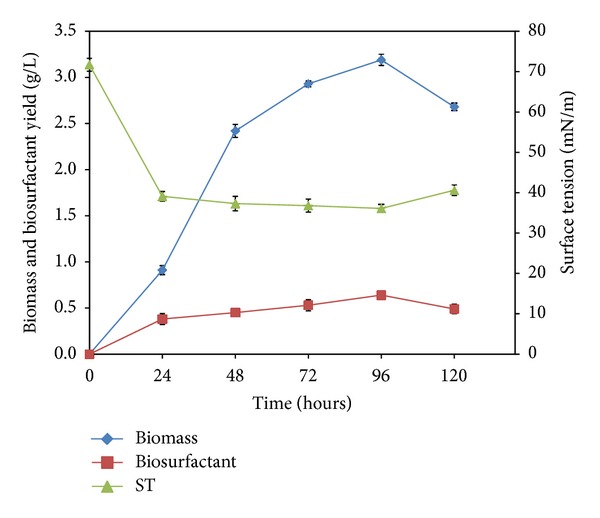
Growth, biosurfactant production, and surface tension profiles of *Bacillus siamensis *grown in MSM with 2% (v/v) crude oil at 30°C and 150 rpm.

Since the isolate was enriched from an oil-contaminated site, preliminary batch fermentation studies were performed in mineral salt medium, Makkar and Cameotra [23], supplemented with 2% (v/v) crude oil as the sole carbon substrate. 24-hour cultures were periodically sampled out to monitor the biomass growth, biosurfactant production, and surface tension. As shown in Figure 2, at 96 h of fermentation maximum biosurfactant yield of 0.64 g/L, maximum biomass yield of 3.2 g/L, and the lowest surface tension of 36.1 mN/m were obtained. The emulsification index (E_24_) of the culture supernatant against hexadecane reached a maximum of 70% at 72 h.

### 3.6. Thin Layer Chromatography

When the plate was sprayed with 0.2% ninhydrin, the biosurfactant component was observed as a single spot on the TLC plate. The observation implied the presence of amino acids in the sample.

### 3.7. Fourier Transform Infrared Spectroscopy

**Figure 3:**
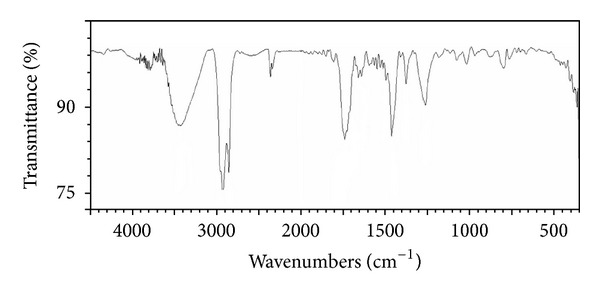
Fourier transform infrared spectra (FTIR) of the biosurfactant produced by *Bacillus siamensis.*

As a result of C–H stretching vibrations and N–H stretching vibrations, a broad absorbance peak (centred around 3433 cm^−1^) with wave numbers ranging from 3600 cm^−1^ to 3100 cm^−1^ was observed (Figure 3). This is typical of carbon-containing compounds with amino groups. Sharp absorbance peaks are observed at 1463 cm^−1^, 1379 cm^−1^, 2955 cm^−1^, and 2854 cm^−1^ and are indicative of aliphatic chains (–CH_3_ and –CH_2_–). These peaks reflect the presence of alkyl chains in the compound. A strong band was also observed at 1741 cm^−1^, 1726 cm^−1^, and 1713 cm^−1^. This is due to a carbonyl group. The presence of C=O bonds causing C=O stretching vibrations leads to absorbance peaks in these regions. The FTIR spectrum implied the production of a lipopeptide biosurfactant.

### 3.8. Stability Analysis of the Biosurfactant

**Figure 4:**
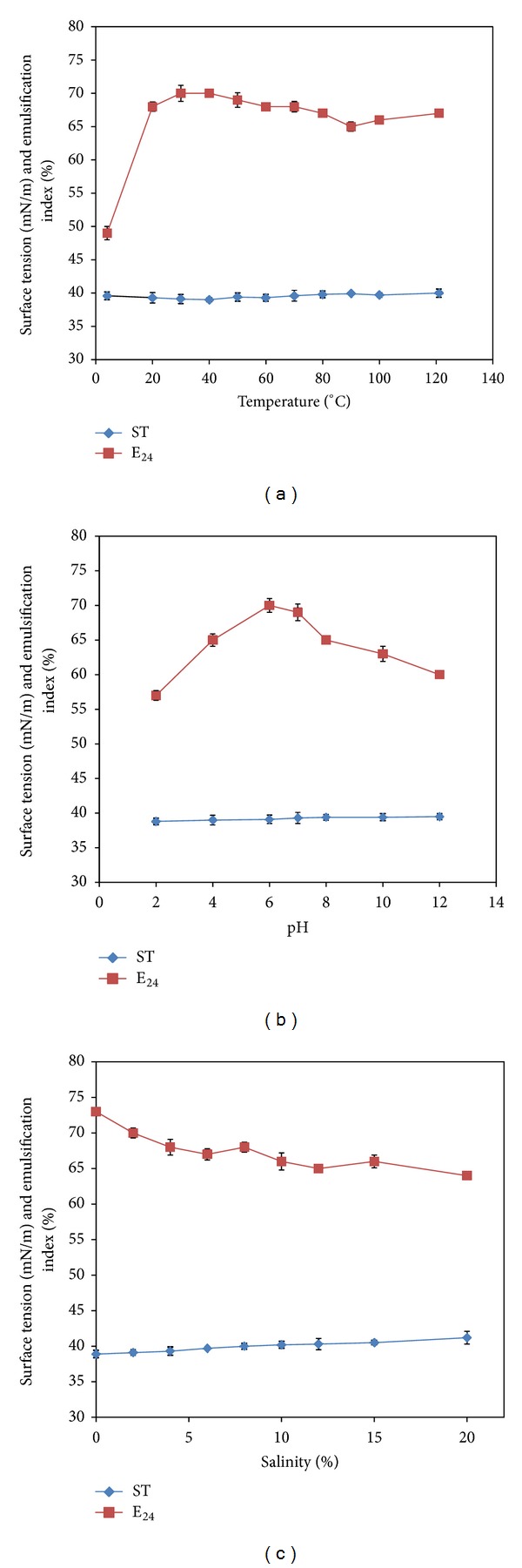
Effect of (a) temperature, (b) pH, and (c) salinity on the stability of biosurfactant.

The stability of the biosurfactant was checked by subjecting the fermentation broth to conditions of high stress that included temperature, pH, and salinity. When the temperature was varied from 0°C to 121°C, both surface tension and emulsification index (E_24_) showed little variation and remained nearly constant at around 39-40 mN/m (Figure 4(a)). Similar results were observed by many researchers [24, 25].

With respect to pH variation from 2 to 12, the values of surface tension were centred around 39 mN/m without large deviations (Figure 4(b)). The emulsification index (E_24_) dipped to lower values (60%), when the pH was increased. Variability in surface tension and emulsification index was not profoundly observed in various studies involving changes in broth pH which is in accordance with the literature [26, 27]. 

The salinity was varied over the range of 0–20%. The effect on surface tension was similar to the effect of pH with largely no changes (Figure 4(c)). The emulsification index (E_24_) decreased to 64% at 20% saline level. The literature study in the past also confirms that many biosurfactants have stable surface activity even at high levels of salinity [27, 28].

### 3.9. Suitability for Microbial Enhanced Oil Recovery

The applicability of the biosurfactant was verified using the sand pack column test [21], while maintaining the column at 30°C, 50°C, and 70°C. The oil recovery was 48% at 30°C. With rise in temperature, the recovery also increased to 55% and 60% at 50°C and 70°C, respectively. Bordoloi and Konwar [29] performed the study at room temperature, 70°C, and 90°C using a sand pack column. They reported oil recovery in the range from 35% to 60% across the temperatures, for various *Bacillus* and *Pseudomonas* strains.

## 4. Conclusions

In the present study, biosurfactant-producing organisms were enriched and isolated from an oil-contaminated site. The morphological, biochemical, and 16S rRNA analysis were performed to identify the organism. This is the first report describing the isolation and use of *Bacillus siamensis* as a biosurfactant producer. During fermentation studies, the isolate was able to produce a lipopeptide biosurfactant, using crude oil as the sole carbon source. The biosurfactant was extracted and partially characterized by using TLC and FTIR spectroscopy to confirm its chemical nature. A maximum biosurfactant yield of 0.64 g/L was obtained at 96 h. The biosurfactant had good surface tension reducing capability, reducing the surface tension to 36.1 mN/m. Stability studies were performed to investigate the effect of extreme variations in temperature, pH, and salinity levels on the surface tension and emulsification capacities of the biosurfactant. In order to examine the suitability of the biosurfactant in enhanced oil recovery processes, sand pack column studies were carried out. To simulate real situations, the column studies were performed at higher temperatures, and the percentage of oil recovery was estimated to be 60%. The organism and its product present a huge potential for use in environmental remediation strategies.

## Figures and Tables

**Table 1:** Examples of microbial biosurfactants.

Microorganism	Biosurfactant
*Torulopsis *sp.	Sophorolipids
*Pseudomonas *sp.	Rhamnolipids
*Rhodococcus erythropolis *	Trehalose lipids and mycolates Sucrose and fructose lipids
*Rhodococcus *sp.	Trehalose lipids
*Candida *sp.	Mannosyl erythritol lipid
*Candida bogoriensis *	Sophorolipid
*Acinetobacter * sp.	Fatty acid, glycerides, and emulsan
*Corynebacterium lepus *	Corynemycolic acids
*Candida petrophilum *	Peptidolipid
*Bacillus subtilis *	Cyclic lipopeptide
*Bacillus licheniformis *	Cyclic lipopeptide
*Candida tropicalis *	Mannan-fatty acid complex
*Corynebacterium hydrocarboclastus *	Proteo-lipid-carbohydrate complex

**Table 2:** Summary of the screening tests for biosurfactant producers.

Isolate nos.	Screening tests					
	OST	BAHT	DCT	CTAB	TGST	ST
	(cm)	(cm)	(cm)	(Green halo)	(+/−)	(mN/m)
RT1	2.0	2.2	0.4	n.o.	+	62.3
RT2	2.5	3.1	1.1	n.o.	−	39.7
RT3	1.3	1.8	0.2	Green halo	+	37.6
RT4	2.4	2.6	1.6	n.o.	−	42.6
RT5	1.7	1.5	1.3	n.o.	−	59.5
RT6	1.0	2.5	0.5	n.o.	+	48.8
RT7	2.7	3.0	1.2	n.o.	−	53.2
RT8	1.4	2.5	0.4	Green halo	+	42.1
RT9	1.7	1.9	0.0	n.o.	−	60.0
RT10	3.0	3.5	1.7	n.o.	−	29.8
RT11	2.1	2.8	0.5	n.o.	−	64.6

OST: oil spreading technique; BAHT: blood agar Hemolysis test; DCT: drop collapse test;CTAB: CTAB agar plate test;TGST: tilting glass slide test; ST: surface tension; n.o.: not observed; “+”: flow observed; “−”: flow not observed.

**Table 3:** Morphological features of the bacterial isolate RT10.

Morphological feature	Observation
Shape	Rod
Colour	Pale white
Size	2–4 mm diameter
Surface	Shiny
Texture	Moist
Edge	Entire
Elevation	Raised
Opacity	Translucent

**Table 4:** Biochemical characteristics of the bacterial isolate RT10.

Biochemical test	Result
Gram staining	Positive
Motility test	Motile
Starch hydrolysis test	Positive
Casein hydrolysis test	Positive
Methyl red test	Negative
Voges Proskauer test	Positive
Citrate utilization test	Negative
Catalase test	Positive
Glucose fermentation	Positive
Endospore staining	Presence of endospore
